# Does childhood trauma predict schizotypal traits? A path modelling approach in a cohort of help-seeking subjects

**DOI:** 10.1007/s00406-021-01373-6

**Published:** 2022-01-04

**Authors:** Julian Max Bernhard Dizinger, Carolin Martha Doll, Marlene Rosen, Michael Gruen, Lukas Daum, Frauke Schultze-Lutter, Linda Betz, Joseph Kambeitz, Kai Vogeley, Theresa Katharina Haidl

**Affiliations:** 1 Department of Psychiatry and Psychotherapy, Faculty of Medicine, University Hospital Cologne, University of Cologne, Kerpener Straße 62, 50937 Cologne, Germany; 2 Department of Psychiatry and Psychotherapy, Medical Faculty, Heinrich-Heine University, Düsseldorf, Germany; 3 Department of Psychology, Faculty of Psychology, Airlangga University, Surabaya, Indonesia; 4 University Hospital of Child and Adolescent Psychiatry and Psychotherapy, University of Bern, Bern, Switzerland; 5 Research Center Jülich, Institute of Neuroscience and Medicine—Cognitive Neuroscience (INM3), Jülich, Germany

**Keywords:** Schizotypy, Childhood trauma and adversities, Trauma and distress scale, Wisconsin schizotypy scales, Path model, Psychosis

## Abstract

Schizotypy constitutes a susceptibility to beneficial and deleterious schizotypal traits, ranging from coping mechanisms to schizotypal personality disorder on a psychosis continuum. Growing evidence indicates a relationship between childhood adversity and trauma and schizotypy. However, the exact influence of childhood adversity and trauma on schizotypy and its relation to sex is not sufficiently understood. Therefore, we investigated sex-adjusted connections between childhood adversity and trauma subdomains (emotional/physical/sexual abuse, emotional/physical neglect) and positive (magical ideation, perceptual aberration) as well as negative schizotypy (physical/social anhedonia). In total, 240 outpatients of the Early Detection and Intervention Centre of the University Hospital Cologne were assessed with the Trauma and Distress Scale for childhood adversity and trauma and the Wisconsin Schizotypy Scales for schizotypy. Path analyses were performed to investigate sex-adjusted correlations. The well-fitting path model of the total sample linked emotional abuse to magical ideation (*p* = 0.03; SE = 0.20) and emotional neglect to social anhedonia (*p *= 0.01; SE = 0.26). In females, physical abuse predicted magical ideation (*p* = 0.01; SE = 0.33), while emotional neglect forecasted physical anhedonia (*p* = 0.03; SE = 0.34) and social anhedonia (*p *= 0.03; SE = 0.32). In males, sexual abuse predicted perceptive aberration (*p* = 0.04; SE = 0.19) and emotional abuse forecasted magical ideation (*p* = 0.03; SE = 0.27). Overall, the significance of sex-specific interrelations between trauma and schizotypy were highlighted. Magical ideation and perceptive aberration occurred prominently in the absence of negative and disorganized schizotypy, thus positive schizotypy could be discussed as a beneficial expression of coping with emotional, physical and sexual abuse. Furthermore, emotional neglect should be addressed particularly to prevent deleterious negative schizotypy in females.

**Trial registration number** (20-1243), **date of registration** (May 19th 2020), retrospectively registered.

**Supplementary Information:**

The online version contains supplementary material available at 10.1007/s00406-021-01373-6.

## Introduction

### Schizotypy

Schizotypy is defined as a latent personality structure [1], a multidimensional construct comprising of positive, negative and disorganized schizotypy dimensions [2], including deleterious as well as beneficial schizotypal traits [3, 4]. In detail, “positive schizotypy” is characterized by disruptions in thought content, perceptual aberration, suspiciousness and potentially benign magical ideation, while “negative schizotypy” covers diminution in experiences including alogia, anergia, avolition, anhedonia, diminished affect and disinterest in others and the world [2, 5]. There are different definitions of schizotypy, each depending on its historical background. In current research, three dominant models are discussed [6–8]: First, the taxonic or quasi-dimensional model by Meehl et al. [8], second and third the two fully dimensional models as described by Eysenck et al. [7] and Clardige et al. [6]. However, all three concepts have in common, that they refer to schizotypy as an individual susceptibility to beneficial and deleterious schizotypal traits, ranging from coping mechanisms to schizotypal personality disorder (SPD) on a psychosis continuum.

### Relationship of schizotypy and SPD

In general, SPD cannot be equalled with schizotypy and it is important to not overlook the sophisticated disparities distinguishing the two concepts [3] (please see Table S2). Both encompass enduring personality traits, but in contrast to the diagnosis SPD listed in the International Classification of Diseases 10th revision (ICD-10) [9], schizotypy is not per se pathological or classified as a disorder. Schizotypy is understood as a multidimensional construct or personality structure, inheriting positive, negative and disorganized dimensions. Studies suggest that latent personality traits intrinsic of schizotypy’s negative dimension might lead to the development of SPD [3]. Though SPD and schizotypy are two different constructs measured by different instruments, they share common ground and overlap phenomenologically [3, 10]. Both of their positive and negative dimensions agree in defining symptoms (such as magical thinking, perceptive aberration and diminished affect) [3].

### Relationship between childhood trauma and schizotypy

Childhood trauma is correlated with an increased likelihood of developing traits of schizotypy, especially with a dose–response relationship with positive features [11]. Particularly paranoid ideation (suspiciousness) and complex social cognitive skills were shown to be affected by trauma exposure in childhood [12]. With respect to the five individual trauma domains (physical, emotional and sexual abuse as well as physical and emotional neglect), all were found to be predictive features of schizotypal traits [13–15]. Despite discrepancies regarding the differential effects of adverse events and trauma subdomains on the development of features of schizotypal traits, the strongest and most consistent effects were found for emotional abuse [11]. The possible pathogenic mechanisms underlying the relationship between impairments in adults with psychotic disorders (or schizotypy) and childhood trauma are still poorly understood [16]. Primarily, the traumagenic neurodevelopment (TN) model [17, 18] postulates that sufficiently severe trauma may contribute to pathological alterations in neurodevelopmental processes, such as changes in the hypothalamic–pituitary–adrenal (HPA) axis [19]. Other hypotheses assume specific neural mechanisms that may be involved, e.g., that abnormal dopaminergic function may be the final pathway linking childhood adversity to psychotic symptoms [20]. It has also been speculated, that the different types of childhood trauma all involve a process of social defeat, which could be an essential link [21]. Although gender differences have been shown in the past to play an important role in the association between aversive childhood experiences and psychosis [22] including the possibility of different mechanistic pathways leading to psychosis in males and females, the association between childhood abuse and schizotypy is less clear [23]. There are conflicting results on potential sex-specific influences of childhood adversity and trauma subdomains on schizotypal traits [11, 24, 25]**.** While emotional abuse was associated with most schizotypal traits in both sexes [11, 24], physical abuse was shown to be associated with positive and negative schizotypal traits only in females [24]. Although females obtained higher scores for sexual abuse, this trauma subdomain did not predict schizotypal traits in the presence of the other subdomains in either males or females [24]. In contrast, Berenbaum and colleagues reported that higher levels of all forms of childhood adversity were associated with higher levels of schizotypal traits in both sexes—although correlations were often low [25]. Thus the role of sex in relationships between different types of childhood trauma and schizotypy are not yet well understood and rarely studied so far.

### Hypothesis

To close this gap, this study aims to investigate the precise influences and interrelations of the individual aspects of childhood trauma on positive and negative schizotypy and its relationship to sex in a large help-seeking sample from the Cologne Early Detection and Intervention Centre (FETZ) [26]. Importantly, we studied schizotypal traits that are not per se regarded as pathological, irrespective of the presence of a schizotypal personality disorder. In light of the current research, we hypothesize that positive and negative schizotypy are mostly linked to emotional abuse in both sexes [11, 24], whereas we expect that physical abuse is linked to both positive and negative schizotypy only in females [24]. No significant link with schizotypy is assumed for sexual abuse [24].

## Method

### Included and excluded patients

The data for this study were collected as part of clinical examinations from the Early Detection and Intervention Centre for Mental Disorders (FETZ) of the Department of Psychiatry and Psychotherapy at the University Hospital of Cologne [26] and are part of the baseline data of an ongoing catamnestic study, which was approved by the ethics committee of the Medical Faculty of the University of Cologne (ID 19-1618_1) and registered at the German Clinical Trials Register (DRKS-ID: DRKS00024469). The trial presented in this article was also approved by the local ethics committee (ID 20–1243).

The sample consisted of *N* = 516 patients who sought help at the FETZ between 2002 and 2010. The patients gave written consent for their data to be used for study purposes. The FETZ is an outpatient service that offers patients of age 18–40 low-threshold access, diagnostic evaluation and assessment, especially for the clinical high-risk for psychosis.

Exclusion criteria were: (1) insufficient information (*n* = 257) and disorders caused by substance abuse and/or inflammatory/ traumatic brain injuries (*n* = 19) (see Fig. S1). As a result, data of 240 patients could be included in the present analyses. Their sociodemographic and clinical data are detailed in Table 1. A comparison between the included and the excluded sample can be found in detail in Table S1.

**Table 1:** Sociodemographic and clinical characteristics of the sample (*n* = 240)

	Total sample (*n* = 240, 100%)	Females (*n* = 99, 41.3%)	Males (*n* = 141, 58.8%)	Statistics *x*^2^_(df)_/U	*P *value
Age (in years), Mean (± SD)	24.73 (± 5.6)	24.58 (± 6.1)	24.84 (± 5.3)	6612.5	0.487
Median (range)	24 (15–50)	23 (15–50)	24 (16–40)		
Partnership, *n* (%)^**§**^	149 (62.1)	90 (90.9)	59 (41.8)	11.984_(1)_	0.001**
Single	98 (40.8)	69 (69.7)	29 (20.6)	9.289_(1)_	0.002**
In steady partnership	51 (21.3)	30 (30.3)	21 (14.9)	8.253_(1)_	0.004**
Married	15 (6.3)	12 (12.1)	3 (2.1)	9.914_(1)_	.002**
Separated	3 (1.4)	2 (2)	1 (< 0.1)	0.810_(1)_	0.368
Education, *n* (%)					
ISCED 1: Primary education	2 (0.8)	1 (1)	1 (< 0.1)	0.064_(1)_	0.801
ISCED 2: Lower secondary education	53 (22.1)	34 (34.3)	19 (13.5)	0.819_(1)_	0.367
ISCED 3: Upper secondary education	154 (64.2)	87 (87.9)	67 (47.5)	0.903_(1)_	0.342
ISCED 4: Post-secondary non-tertiary education	18 (7.5)	8 (8.1)	10 (7.1)	0.082_(1)_	0.775
ISCED 5: Short-cycle tertiary education	23 (9.6)	9 (9.1)	14 (9.9)	0.047_(1)_	0.828
ISCED 6: Bachelor’s or equivalent level	10 (4.2)	5 (5.1)	5 (3.5)	0.330_(1)_	0.566
Occupation, n (%)					
No occupation	47 (19.6)	18 (18.2)	29 (20.6)	0.210_(1)_	0.647
Current occupation and apprenticeship	136 (56.7)	64 (64.6)	72 (51.1)	4.370_(1)_	.037*
Risk criteria for the development of a psychotic first manifestation fullfilled, n (%)					
Basic Symptom criteria	88 (36.7)	40 (40.4)	48 (34)	1.014_(1)_	0.314
Ultra High Risk criteria	25 (10.4)	12 (12.1)	13 (9.2)	0.525_(1)_	0.469
Basic Symptoms & Ultra High Risk criteria	15 (6.3)	7 (7.1)	8 (5.7)	0.194_(1)_	0.660
No criteria met	142 (59.2)	54 (54.5)	88 (62.4)	1.490_(1)_	0.222
Clinical characteristics^§^					
Clinical high-risk criteria met, *n* (%)	111 (46.3)	50 (50.5)	61 (43.3)	1.227_(1)_	0.268
Clinical High Risk but no ICD-10 diagnosis, *n* (%)	77 (31.3)	36 (34.4)	41 (29.1)	1.417_(1)_	0.234
Any current ICD-10 diagnosis, *n* (%)^**§**^	63 (40.8)	35 (14.6)	28 (19.7)	0.819_(1)_	0.366
F2 Schizophrenia, schizotypal and delusional disorders^**a**^	28 (11.7)	13 (13.1)	15 (10.6)	0.351_(1)_	0.554
F21 Schizotypal personality disorder	10 (4.2)	6 (6)	4 (2.8)	1.866_(1)_	0.172
F3 Mood (affective) disorders^**b**^	51 (21.3)	20 (20.2)	31 (22)	0.111_(1)_	0.739
F4 Neurotic, stress-related and somatoform disorders^**c**^	25 (10.4)	16 (16.2)	9 (6.4)	0.985_(1)_	0.321
F5 Behavioral syndromes associated with physiological disturbances and physical factors^**d**^	2 (< 0.1)	1 (< 0.1)	1 (< 0.1)	0.064_(1)_	0.801
F6 Disorders of adult personality and behavior^**e**^	8 (< 0.1)	4 (< 0.1)	4 (2.8)	.261_(1)_	.609
Other current diagnosis, *n* (%)^**f**^	4 (< 0.1)	2 (< 0.1)	2 (< 0.1)	0.129_(1)_	0.720

ISCED 1–6 = International Standard Classification of Education Level pursuant to the 36th General Conference of the United Nations Educational, Scientific and Cultural Organization

ICD-10 = International Classification of Diseases, 10th Revision

^*^p < 0.050, **p < 0.000

^§^Multiple group memberships possible

^a^Schizotypal disorder (4.2%), schizophrenia (7.5%)

^b^Depressive disorder (16.3%), recurrent depressive disorder (1.7%), bipolar disorder (0.8%), manic episode (0.4%)

^c^Phobic disorder (5.4%), reaction to severe stress and adjustment disorders (3.3%), obsessive–compulsive disorder (1.3%), somatoform disorders (0.8%), other anxiety disorders (0.8%)

^d^Sleep disorders not due to a substance or known physiological condition (0.4%), psychological and behavioral factors associated with disorders or diseases classified elsewhere (0.4%)

^e^Specific personality disorder (2.9%), impulse disorders (0.4%)

^f^Problems related to life management difficulty (1.7%)

^a^^−^^f^Information listed in the subheading in reference to total sample of *n* = 240

*N* = 111 (46.3%) of the included patients met clinical high-risk criteria for psychosis according to the ultra-high risk and/or the basic symptom criteria as assessed with the Structured Interview for Prodromal Symptoms [27] and the Schizophrenia Proneness Instrument, Adult Version [28] (see Table 1). Furthermore, *n* = 63 (40.8%) included patients were diagnosed with an ICD-10 listed mental disorder (see Table 1), with mood and affective disorders accounting for the largest proportion (21.3%), followed by schizophrenia, schizotypal and delusional disorders (11.7%). Schizotypal disorder specifically was present in *N* = 10 patients (4.2%) (see Table 1**)**. *N* = 77 (31.3%) of included patients met Clinical High Risk criteria in the absence of a mental disorder.

### Assessments

Clinical assessments were conducted by trained and experienced psychologists and psychiatrists from FETZ and included routine clinical anamnesis, and assessment of sociodemographic information and mental diagnoses according to the International Classification of Diseases (ICD-10). The clinical assessments were complemented and supported by a variety of questionnaires, including the Wisconsin Schizotypy Scales [29, 30] and the Trauma and Distress Scale [27]. If recommended by the respective guidelines for a certain disorder, laboratory tests and magnetic resonance imaging were performed to rule out somatic causes of mental health problems.

To assess childhood adversity and trauma, the 43-item Trauma and Distress Scale for the self-report assessment of the five trauma domains emotional abuse (five items) and neglect (five items), physical abuse (five items) and neglect (five items) as well as sexual abuse (five items) was used [29]. Patients are requested by the questionnaire to give the frequency of single childhood adversities and trauma on a five-point Likert-scale ranging from 0 = never to 4 = always [29]. Thereby, the instructions give the evaluation period as follows: “Many questions refer to ‘when you were young’: this means the period of your life when you were growing up and before you left school.” [29]. In addition, they clarify the use of the word ‘parents’ as “the adults who had the main responsibility for your upbringing as a child and teenager” [29].

Overall, the Trauma and Distress Scale has demonstrated good validity, reliability and robust cut-offs [29].

Positive and negative schizotypy were assessed by the four Wisconsin Schizotypy Scales [30, 31]: the two positive scales ‘perception aberration’ (21 items) and ‘magical ideation’ (20 items), as well as the two negative scales ‘revised social anhedonia’ (40 items) and ‘physical anhedonia’ (50 items) [30–32]. The items give statements that can be affirmed or negated. The Wisconsin Schizotypy Scales have demonstrated strong test–retest reliability [33, 34] as well as high internal consistency [35].

### Data analyses

Statistical analyses were conducted using the SPSS version 25 [36]. Path analysis was performed with Open Source “R” version 4.0.2, utilizing the “lavaan” package [37, 38]. After excluding all patients with less than 99% completed questionnaires, the remaining missing questionnaire items (overall 0.65% of 6.000 individual Trauma and Distress Scale items and 0.9% in 32.880 individual Wisconsin Schizotypy Scales items in finally 240 included patients) were replaced by mean imputation.

As the respective domains or scales (emotional neglect and abuse, physical neglect and abuse as well as sexual abuse for the Trauma and Distress Scale; magical ideation, perceptive aberration, physical and social anhedonia for the Wisconsin Schizotypy Scales) are well represented by their sum scores, it was not necessary to formulate latent variables in the sense of a structural equation model. Therefore, sum scores were directly included as observed variables in the path analysis. A step-by-step approach was chosen to first explore the relevant links between the listed domains and scales, calculating bivariate regressions and, second, testing for possible sex effects. The path-analysis model was postulated classifying all five Trauma and Distress Scale domain scores as endogenous variables of the type x, examining every possible path interrelating with the four Wisconsin Schizotypy Scales, classified as endogenous variables of the type y. In addition, to control for possible confounding effects, sex was included as well. Thus, a total number of 29 paths were investigated in an over-identified model. Considering the total sample of *n* = 240, the number of paths proved to be too high for the sample size as the model fit parameters failed to meet the required thresholds [39, 40]. In general, literature suggests a patients: paths ratio of five to ten patients per calculated path [39, 40].

For the evaluation of model fit, an abundance goodness-of-fit indices are available, whose estimates depend on factors such as small-sample bias, degrees of freedom, model misspecification, estimation method effects and more [41]. The goodness-of-fit indices strictly follow the recommendations and guidelines of Hu and Bentler et al. (1999), Kline et al. (2005) as well as Hooper et al. (2008)—this includes: The *χ*2-test, the Comparative Fit Index (CFI), the Tucker Lewis index (TLI), the Root Mean Square Error of Approximation (RMSEA) and Standardized Root Mean Square Residual (SRMR) [42–44]. Furthermore, Maximum Likelihood estimation was used. To certify a good model fit, results of the *χ*2-test should be *p* ≥ . 05*,* CFI and TLI should range from 0.97 to 1.0, the latter representing optimal fit. In addition, RMSEA and SRMR should rank from 0 to 0.05, in which 0 is optimal [41, 42, 44]. Following Hu and Bentler’s (1999) ‘Two-Index Presentation Strategy’, RMSEA and SRMR were reported [42, 44].

To meet the model fit criteria, a reduction of analyzed paths was necessary to diminish misspecification and small-sample bias. For this purpose, we excluded sex from the model to build a more parsimonious just-identified path model, for which a good model fit was predicted [45]. This allowed to still account for sex effects, applying the final model in an iterative manner first to the total sample (*n* = 240) and second to sex-specific samples (*n* = 99 female, *n* = 141 male). Subsequently, the observed effects of the three models were compared as well as intercorrelations for all Trauma and Distress Scale domains and Wisconsin Schizotypy Scales tested. In addition, a Mann–Whitney *U *Test was calculated respecting Kolmogorov–Smirnov *p* < 0.05 and *p* < 0.00 to revise for potential significant distribution disparities in the female and male sample.

## Results

### Parameter value of trauma and schizotypal traits

With regard to childhood adversity and trauma and the Wisconsin Schizotypy Scales males and females only differed significantly in the trauma domain sexual abuse (*U* = 5299.50; *p* < 0.00; *Z* = − 3.92) and the Wisconsin Schizotypy subscale perceptive aberration (*U* = 5769.00; *p* < 0.05; *Z* = − 2,32), with females showing higher scores (see Table 2). Other trauma domains or Wisconsin Schizotypy subscales revealed no significant divergencies between both sex categories.

**Table 2:** TADS and WSS sum scores of sex-adjusted samples in a Mann–Whitney *U *Test

Sum score	Female (*n* = 99, 41,25%) Mean / SD	Male (*n* = 141, 58,75%) Mean / SD	Mann–Whitney *U*	Significance
TADS				
Emotional neglect	7.40 ± 4.41	6.93 ± 4.11	6555.50	0.422
Emotional abuse	5.75 ± 4.75	4.99 ± 4.45	6334.00	0.221
Physical neglect	4.07 ± 2.62	4.02 ± 2.65	6948.50	0.953
Physical abuse	1.79 ± 2.65	1.82 ± 2.51	6782.50	0.696
Sexual abuse	2.43 ± 4.70	0.90 ± 2.75	5299.50	0.000**
WSS				
Magical ideation (MagId)	5.06 ± 3.37	4.31 ± 3.46	5989.00	0.060
Perceptual aberration (PercAb)	3.37 ± 3.71	2.48 ± 3.16	5769.00	0.020*
Physical anhedonia (PhAnh)	16.27 ± 8.37	17.94 ± 8.51	6255.00	0.171
Social anhedonia (SocAnh)	14.92 ± 6.71	15.12 ± 6.77	6843.50	0.797

^*^p < 0.050, **p < 0.000

### Sex-specific and cross cutting path analysis

**Fig. 1:**
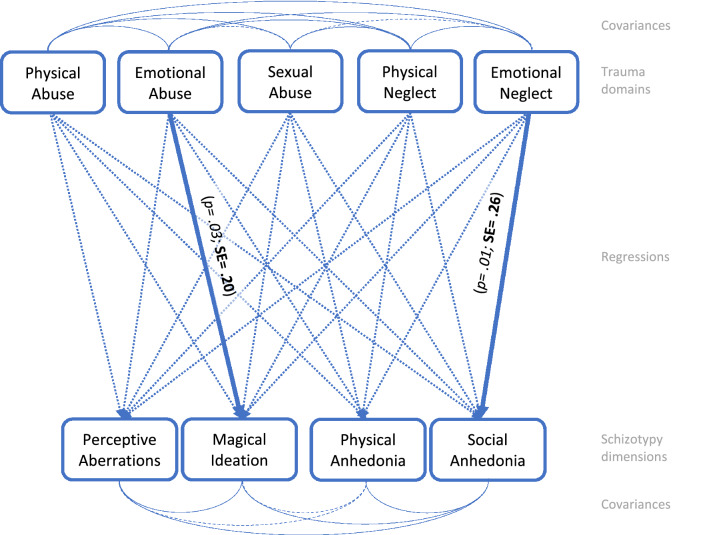
Path analysis of the total sample, *n* = 240. Path analysis model of the calculated regressions between trauma domains and schizotypy scales. Dashed arrows present non-significant paths (*p > 0.050*). Highlighted arrows display significant paths (*p < 0.050)* with standardized estimates in bold script, *p *values in italics. Model fit: RMSEA: 0.000; SRMR: 0.000; For in-depth review of the intercorrelation analysis of all trauma domains and schizotypy dimensions see Table 3

**Fig. 2:**
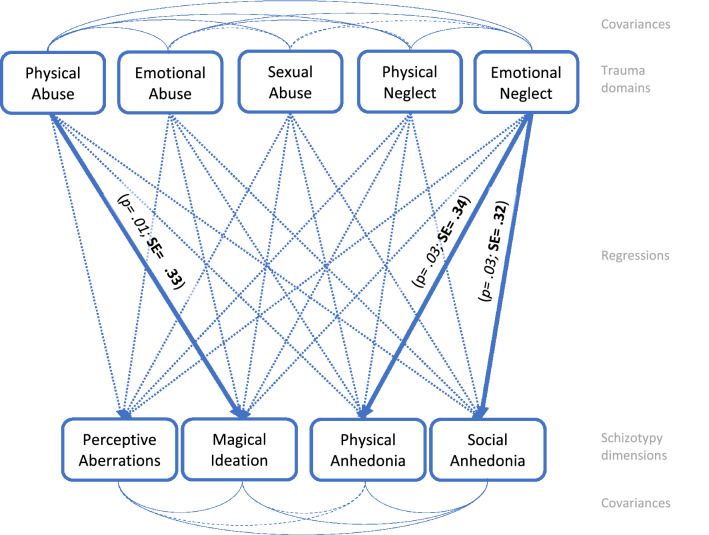
Path analysis, female sample, *n* = 99. Path analysis model of the calculated interrelations between trauma domains and schizotypy scales. Dashed arrows present non-significant paths (*p > 0.050*). Highlighted arrows display significant paths (*p < 0.050)* with standardized estimates in bold script, *p *values in italics. Fit: RMSEA: 0.000; SRMR: 0.000; For in-depth review of the intercorrelation analysis of all trauma domains and schizotypy dimensions see Table S2 in the supplementary material

**Fig. 3:**
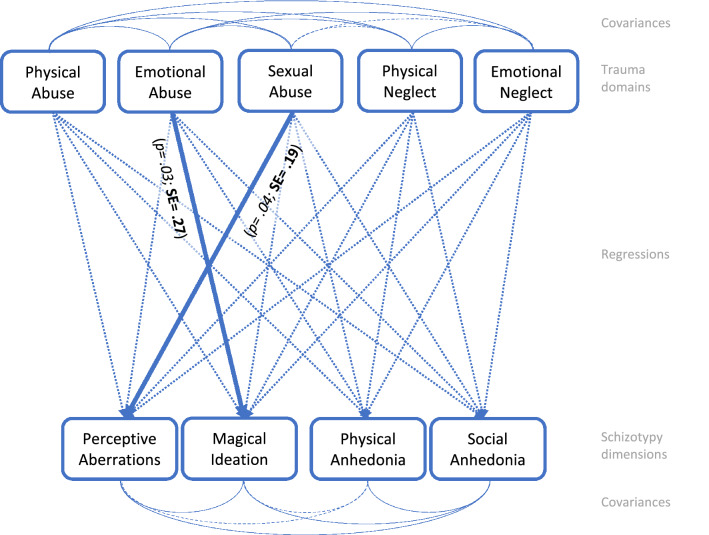
Path analysis, male sample, *n* = 141. Path analysis model of the calculated interrelations between trauma domains and schizotypy scales. Dashed arrows present non-significant paths (*p > 0.050*). Highlighted arrows display significant paths (*p < 0.050)* with standardized estimates in bold script, *p *values in italics. Fit: RMSEA: 0.000; SRMR: 0.000; For in-depth review of the intercorrelation analysis of all trauma domains and schizotypy dimensions see Table S3 in the supplementary material

The three saturated models of the total sample as well as in males and females yielded good model fits (see Figs. 1, 2, 3). In the total sample, emotional abuse was linked to magical ideation and emotional neglect to social anhedonia (see Fig. 1). The latter link was also found in the female subsample, in which it was substituted by an additional significant path from emotional neglect to physical anhedonia; magical ideation, however, was associated with physical abuse rather than emotional abuse in this sample (see Fig. 2). No link between childhood adversity and trauma and negative schizotypy was found in the male subsample (see Fig. 3); rather the association between emotional abuse and magical ideation of the total sample became significant in males, too, and was substituted by a significant path between the rarely occurring sexual abuse (see Table 2) and perceptive aberration (see Fig. 3). A complete table of regressions (see Table 3 and Tables S4–5) as well as a comprehensive analysis of intercorrelations of the Trauma and Distress Scale domains and Wisconsin Schizotypy Scales can be found in the supplementary material (see Tables S6–8).

**Table 3:** Total sample (*n* = 240) path-analysis regression, covariance, variance and R-Square data

Regressions	Estimate	Std. Err	*z* value		*P*( >|*z*|)	Std. all	
Perceptual aberration							
Emotional neglect	0.044	0.077	0.565		0.572	0.054	
Emotional abuse	0.098	0.070	1.405		0.160	0.131	
Physical neglect	0.160	0.104	1.537		0.124	0.124	
Physical abuse	0.105	0.107	0.980		0.327	0.079	
Sexual abuse	0.063	0.063	1.004		0.315	0.070	
Magical ideation							
Emotional neglect	− 0.064	0.079	− 0.813		0.416	− 0.079	
Emotional abuse	0.152	0.071	2.139		0.032	0.203	
Physical neglect	0.138	0.106	1.296		0.195	0.106	
Physical abuse	0.149	0.109	1.366		0.172	0.111	
Sexual abuse	0.017	0.064	0.271		0.787	0.019	
Physical anhedonia							
Emotional neglect	0.510	0.201	2.544		0.011	0.255	
Emotional abuse	− 0.255	0.181	− 1.410		0.159	− 0.138	
Physical neglect	− 0.241	0.271	− 0.890		0.374	− 0.075	
Physical abuse	0.238	0.278	0.856		0.392	0.072	
Sexual abuse	0.028	0.164	0.170		0.865	0.012	
Social anhedonia							
Emotional neglect	0.287	0.155	1.853		0.064	0.180	
Emotional abuse	0.062	0.140	0.443		0.658	0.042	
Physical neglect	0.237	0.209	1.136		0.256	0.093	
Physical abuse	0.006	0.214	0.027		0.979	0.002	
Sexual abuse	0.110	0.126	0.869		0.385	0.061	
Covariances:	Estimate	Std. Err	*z* value		*P*( >|*z*|)	Std. all	
Perceptual aberration							
Magical ideation	5.567	0.768	7.253		0.000	0.530	
Physical anhedonia	2.457	1.734	1.417		0.157	0.092	
Social anhedonia	5.039	1.371	3.676		0.000	0.244	
Magical ideation							
Physical anhedonia	1.218	1.762	0.692		0.489	0.045	
Social anhedonia	3.266	1.374	2.378		0.017	0.155	
Physical anhedonia							
Social anhedonia	39.406	4.291	9.184		0.000	0.736	
Variances:	Estimate	Std. Err	*z *value		*P*( >|*z*|)	Std. all	
Perceptual aberration	10.310	0.941	10.954		0.000	0.887	
Magical ideation	10.710	0.978	10.954		0.000	0.910	
Physical anhedonia	69.417	6.337	10.954		69.417	0.970	
Social anhedonia	41.288	3.769	10.954		0.000	0.914	
R-Square:	Estimate						
Perceptual aberration	0.113						
Magical ideation	0.090						
Physical anhedonia	0.030						
Social anhedonia	0.086						

## Discussion

The aim of this study was to examine the possible predictive potential of childhood adversity and trauma subdomains regarding schizotypal traits in females and males. We had hypothesized that emotional abuse plays the most important role in positive and negative schizotypy in both sexes. This could only be partially confirmed for positive schizotypy (magical ideation) in both sexes. Furthermore, negative schizotypy (social anhedonia) and emotional neglect were positively associated in both sexes. Besides cross-sex relationships, there also seemed to be sex-specific associations. Our hypothesis that physical abuse in females is associated with positive and negative schizotypy was also partially confirmed. Thus, in females, a connection between magical ideation and physical abuse became significant, while in males an association between magical ideation and emotional abuse was shown. In addition, a correlation between negative schizotypy (both physical and social anhedonia) and emotional neglect was revealed in females. Our third assumption was not confirmed. Although sexual abuse was unrelated to schizotypy in females, it was associated with positive schizotypy (perceptive aberration) in males, despite sexual abuse being reported less frequent in these. In summary, the link between abuse and positive schizotypy’s magical ideation is particularly noteworthy as it was depicted in all three model calculations. However, the type of abuse appeared to be sex-specific.

### Similarities and differences in the impact of childhood adversity and trauma on SPD and schizotypy

With regard to SPD, a review by Velikonja et al. [11] demonstrated that all forms of childhood adversity and trauma were associated with SPD and that these associations were particularly strong with positive features of SPD. In our study, this applied for males in whom emotional abuse predicted magical ideation and sexual abuse forecasted perceptive aberration. In females, physical abuse was linked to positive schizotypy solely by magical ideation, while emotional neglect was linked to both negative dimensions, physical and social anhedonia. However, the comparability with the SPD review [8] is limited because our sample consisted of young help-seeking patients from an early detection of psychosis service and not specifically recruited patients with an SPD.

Focusing on schizotypy distinct from SPD, emotional abuse and neglect were reported to be especially powerful predictors for schizotypal traits [13, 24, 46]. This is in line with our results showing that emotional abuse and neglect were associated with positive and negative schizotypy across both sexes. Earlier, it was discussed if the multifarious consequences of abuse and neglect on the domains of schizotypy might be explained by different effects of each type of adversity on the developing brain, with neglect being associated with more serious cognitive and psychosocial deficits [47]. Moreover, a study by Bentall et al. (2008) suggested a link between victimisation due to abuse to an explanatory bias and negative self-esteem, which might ultimately mediate deleterious paranoid beliefs [48]. The authors postulated that individuals affected by childhood adversity and trauma tend to locate the cause of their distress in external sources, which could be objectified as deviations from reality [48]. Paranoid beliefs were also discussed to cause impaired social functioning in individuals on schizotypy-level [49]. These results underline the complexity of the relationship between childhood adversity and trauma and SPD as well as schizotypy, and indicate that the manifold ways of dimensionality of SPD or schizotypal traits are still not fully understood.

### Benign and ‘happy’ schizotypal traits

Positive schizotypy appears to inherit benign or ‘happy’ schizotypal traits [50–52]. It has been postulated that [50] individuals referred as "happy schizotypes" [52] with considerably high positive schizotypy and frequently repeated psychotic experiences, but less negative/ disorganized schizotypal traits compared to the general population, are not only "not sick" but actually benefit from these experiences and are “healthier”. For example, higher levels of positive schizotypy showed to be associated with personal well-being, flexible and unconventional thinking (including creativity), and favorable personality traits and psychological characteristics (e.g., openness to experience, inclination toward fantasy [50]). Remarkably, an increase in creativity and spiritual, religious or esoteric beliefs associated with magical ideas was described [3, 50]. Furthermore, Grant et al. supported the verisimilitude of life-enhancing ‘happy schizotypes’ as a part of positive schizotypy, manifested in the increased occurrence of psychosis-like experiences in non-schizophrenic individuals under stress [53]—thereby increasing their cognitive functioning and organization of thought [50]. In light of our results, this might be valid for females presenting high magical ideation scores incidental to physical anhedonia on the one hand and males reaching significant values in perceptive aberration following sexual abuse as well as magical ideation after emotional abuse on the other hand (see Fig. 1–3).

### Schizotypal traits and psychosis-spectrum disorders

It was previously reported that the different childhood adversity and trauma subdomains seem to have an individual influence on the development of different mental disorders and their functional outcome [54]. Emphasizing the importance of schizotypy in psychosis, Grant et al. quoted schizotypy being “the most influential, comprehensive psychological construct in schizophrenia research” [51, 55]. In the effort of detecting individuals at-risk for psychosis, Schultze-Lutter et al. substantiated the hypothesis that certain schizotypal traits aggravate the proneness for psychosis [3]. This was particularly true for physical anhedonia, which was shown to be predictive for the transition to psychosis of ultra high-risk and clinical high-risk states [3, 56, 57]. That is in line with results of our female sample, where emotional neglect was linked significantly to physical anhedonia. However, it should be kept in mind that our sample was a young, help-seeking sample, and not exclusively patients with an increased risk of psychosis. But, adhering to the idea of identifying individuals who have an increased risk for developing psychosis as early as possible, this finding suggests that researchers and clinicians should be particularly considerate when dealing with women who report emotional neglect. In addition, equally strong associations were detected in the female sample regarding emotional neglect and social anhedonia. Yet this association has to be examined with caution: Multiple studies revealed social anhedonia being heavily influenced by heredity, therefore, the impact of emotional neglect might be biased [10, 58, 59]. Further studies disclosed that negative schizotypy seems to play a key role in psychosis-spectrum disorders [51, 55]. Interestingly, in our data, neglect was always positively correlated with negative schizotypy—however, any type of abuse was always associated with positive schizotypy. In line with this, an earlier study reported that abuse was only found to be associated with positive schizotypy in patients with psychosis, while neglect was related to both schizotypy domains [60]. A recent study of similar sample size and methodology in patients with bipolar disorder also reported an association of emotional and physical abuse and total scores in the Peters Delusion Inventory, a self-report measure of delusional beliefs, but not the presence of psychotic features [61]. The link between childhood adversity and trauma and increased delusional beliefs was not operated through cannabis abuse [61], indicating that the exclusion of substance misusing patients likely had no effect on the overall results of our study.

### Sex-specific peculiarities of the associations between schizotypal traits and childhood adversity and trauma

Miettunen et al. conducted a meta-analysis, finding diverging expression of single SPD features in males and females [62]. Consistent with these findings, our analyses also revealed sex-specific differences in schizotypy according to traumatic experiences. In detail, a higher expression of positive schizotypy in men and a stronger occurrence of negative schizotypy in women was found after childhood adversity and trauma. Analogously to schizophrenia patients, this might be explained by sex-related metabolic differences (for example, in cortisol release) [63]. However, studies by Walter et al. [54] and Grant et al. [53] were not able to demonstrate such differences in women and men with schizotypy. Moreover, Berenbaum et al. [25] emphasized that childhood adversity and trauma was more strongly linked to general SPD features in men than in women. On the one hand, Berenbaum et al.’s research is contrary to our results where more and also stronger associations between childhood adversity and trauma and schizotypy were found in females; on the other hand, their sample of community patients (*n* = 75) differs prominently from our clinical sample (*n* = 240) [13]. In line with the work of Toutountzidis et al. (2018), the women in our sample scored significantly higher in sexual abuse, but we only found a significant association between sexual abuse and positive schizotypy in men, while Toutountzidis et al. observed no significant association between sexual abuse and any schizotypal trait [24]. Overall, these results support the assumption that sex-specific peculiarities, may underlie trauma-schizotypic associations.

Interestingly, although our intercorrelation analysis revealed that, across both sexes, all childhood adversity and trauma domains but sexual abuse were significantly associated. Focusing on the Wisconsin Schizotypy Scales, positive schizotypy interrelates with negative schizotypy’s social anhedonia in both sexes, but never with physical anhedonia (see Figs. 1–3, Tables 3 and S6–8). Keeping in mind that not only childhood adversity and trauma is a source for schizotypy, the influence of inheritance on social anhedonia might be prominent in our sample [10, 58, 59].

### Strengths and limitations

Major strength of our study is the young help-seeking sample from our service, which was not recruited according to specific study criteria and thus might reflect everyday conditions in an early detection center. A further strength of our work is the number of patients in relation to the observed paths in the sample, leading to a stronger test power. At least five, better ten patients should be included per observed path [39, 40]. This patients-to-paths ratio is exceeded sufficiently in the model of the total sample (12:1 ratio) and still met in the sex-specific models (ratio in males: 7:1; ratio in females: 5:1). With regard to the study design, choosing a path analysis to explore the trauma and schizotypy relations with focus on sex disparities is unique in this subject of research.

However, some limitations have to be addressed. As most childhood adversity and trauma assessments such as the Trauma and Distress Scale, retrospectively assessed childhood adversity and trauma, there is the risk of a ‘recall bias’ depending on the individual’s current mental health [64]. Another possible limitation is the missing enclosure of factors such as the age at onset, the frequency and the severity of the exposure to/ experiences of childhood adversity and trauma. Moreover, it is important to consider that childhood adversity and trauma may have different detrimental effects on individuals with impacts on brain development, cognition, interpersonal behavior and clinical symptoms. Additional unmeasured variables, such as genetic risk and neighborhood environmental factors, may also account for aspects of observed associations [65]. Furthermore, it should be noted that interestingly the TADS sum score was higher in the group included into the analyses than in the excluded patients from the sample. Another point that should be critically noted is that the data set is an extract from a larger sample. However, in order not to exceed the permissible number of paths/variables [39, 40] for such an analysis and also not to have to accept a reduction of the sample due to possible missings in other variables, we have limited ourselves to the number of variables we have chosen.

Additionally, another possible limitation is the diminished informative value of the RMSEA fit index, as it is based on non-centrality parameters and closely linked to the relation of degrees of freedom in a structural equation model (SEM) [66]. It is important to consider that path model analysis being a SEM subset, only comprises directly observable variables. If the number of estimated paths in such path models is not greater than the number of observed paths, RMSEA has to be zero [66–69]. Nonetheless, the multivariate statistical modeling conducted by SEM-syntax is regarded as state-of-the-art [38, 69]. Also, selection bias effects have to be discussed. In comparison to the excluded patients (*n* = 276), the included patients (*n* = 240) presented higher mean Wisconsin Schizotypy Scales ratings and lower mean Trauma and Distress Scale scores (see Table S1). Different selection criteria might produce deviating results. However, the mean values of the excluded sample might be biased, as we excluded 257 of the total 276 excluded patients solely due to insufficient Wisconsin Schizotypy Scales and Trauma and Distress Scale information, to comply with the requirement for a parsimonious analysis and to minimise the impact of participation or non-response bias [70]. Lastly, the work of Etain et al. [61] leaves room for a discussion of further studies with a broader participant spectrum and revised, less strict exclusion criteria. Using a similar quasi-dimensional path-analysis, their study design allowed for the inclusion of patients which affirmed the misuse of cannabis—one of the exclusion criterions in our recruiting process [61]. However, the results validate our findings concerning the effects of emotional and physical abuse on schizotypal traits.

### Clinical application and implications for the future

The knowledge gained from this study seeks to foster an in-depth understanding of the relationship of childhood adversity and trauma and schizotypy’s beneficial and defective potentials. Our work has provided valuable insights into the relationships between specific childhood adversity and trauma domains and schizotypal traits, detailing their association with sex in a young adult help-seeking sample from an early detection of psychosis service as one of the only studies in detail. For clinical practice, our results suggest that trauma experiences should be considered in a more differentiated way for each sex. For example, in men who have experienced sexual and/ or emotional abuse, special attention should be paid to positive schizotypal features (perceptive aberrations/ magical ideations). Here, further detailed longitudinal studies should clarify whether the positive schizotypal traits correspond to a kind of resilience-promoting "positive" coping after abuse experiences, or rather to a "negative" coping in the sense of the development of paranoid traits up to the development of a SPD or psychosis. In the case of women who have experienced physical abuse, increased attention should be paid to the development of magical ideations. Again, the point made previously regarding coping strategies is applicable. Overall, a positive coping strategy through "happy" schizotypy should be supported by the clinician, whereas a negative coping strategy through "negative" schizotypy should be more of an indication for psychotherapeutic treatment. In addition, lower-threshold psychotherapeutic treatment should be considered for women who have experienced neglect (both physical and emotional), as there was an association with "negative" schizotypal traits in our results and preliminary work [50, 71–74] suggests that these may be more likely to have psychopathological effects.

## Supplementary Information

Below is the link to the electronic supplementary material.

Supplementary file1 (DOCX 194 KB)

## Data Availability

The data that support the findings of this study are available from the corresponding author, [JD], upon reasonable request.

## Code availability

The data that support the findings of this study are available from the corresponding author, [JD], upon reasonable request.
