# Supplementary material for: Does childhood trauma predict schizotypal traits? A path modelling approach in a cohort of help-seeking subjects
Source: Eur Arch Psychiatry Clin Neurosci. 2022 Jan 4;272(5):909–22. doi: 10.1007/s00406-021-01373-6 (PMC9279245; doi:10.1007/s00406-021-01373-6)
Supplement: Supplementary file 1 — Supplementary file1 (DOCX 194 KB) [file 406_2021_1373_MOESM1_ESM.docx]

**Supplementary Material**

**to**

**Does childhood trauma predict schizotypal traits? A path modelling approach in a cohort of help seeking subjects.**

**by**

Julian Max Bernhard Dizinger^a^, Carolin Martha Doll^a,b^, Marlene Rosen^a^, Michael Gruen^a^, Lukas Daum^a^, Frauke Schultze-Lutter^b,c,d^, Linda Betz^a^, Joseph Kambeitz^a^, Kai Vogeley**^a,e^** und Theresa Katharina Haidl^a^

*^a^ Department of Psychiatry and Psychotherapy, Faculty of Medicine and University Hospital Cologne, University of Cologne, Cologne, Germany*

*^b^ Department of Psychiatry and Psychotherapy, Medical Faculty, Heinrich-Heine University, Düsseldorf, Germany*

*^c^ Department of Psychology and Mental Health, Faculty of Psychology, Airlangga University, Surabaya,Indonesia
^d^ University Hospital of Child and Adolescent Psychiatry and Psychotherapy, University of Bern, Bern, Switzerland
^e^ Research Center Jülich, Institute of Neuroscience and Medicine – Cognitive Neuroscience (INM3), Germany*

| **Table S1:** *Clinical comparison of the included and excluded sample.* | | | |
| --- | --- | --- | --- |
|  | **Included patients** | **Excluded patients** | **Comparison^1^** |
| **n** | 240 | 276 |  |
| **females/males** | 99/141 | 115/161 | x^2^=.009 Cramer-V=.004 p=.924 |
| **mean age (sd)** | 24.72 (±5.6) | 25.57 (±5.6) | U= 34227.5 Z=.804 p=.422 Rosenthal’s r=.035 |
| **mean TADS** | 19.75 (±12.8) | 21.32 (±12.1) | U=2970.5 Z=-8.340 p<.001 Rosenthal’s r=-.37 |
| **mean WSS** | 98.76 (±86) | 97.95 (±85.9) | U=9705.5 Z=-8.172 p<.001 Rosenthal’s r=-.36 |
| **Education, n (%)** |  |  |  |
| ISCED 1: Primary education | 2 (0.8) | 10 (3.6) | x^2^=4.399 Cramer-V=.092 p=0.36 |
| ISCED 2: Lower secondary education | 53 (22.1) | 23 (8.3) | x^2^=19.325 Cramer-V=.194 p=.000 |
| ISCED 3: Upper secondary education | 154 (64.2) | 40 (14.5) | x^2^=135.011 Cramer-V=.512 p=.000 |
| ISCED 4: Post-secondary non-tertiary education | 18 (7.5) | 44 (15.9) | x^2^=8.654 Cramer-V=.130 p=.003 |
| ISCED 5: Short-cycle tertiary education | 23 (9.6) | 125 (45.3) | x^2^=80.012 Cramer-V=.394 p=.000 |
| ISCED 6: Bachelor’s or equivalent level | 10 (4.2) | 5 (2.1)) | x^2^=2.523 Cramer-V=.112 p=.070 |
| **Risk criteria for the development of a psychotic first manifestation, n (%)** |  |  |  |
| Basic Symptoms | 88 (36.7) | 181 (65.6) | x^2^=43.004 Cramer-V=.289 p=.000 |
| Ultra High Risk | 25 (10.4) | 137 (49.6) | x^2^=91.683 Cramer-V=.422 p=.000 |
| Basic Symptoms and Ultra High Risk | 15 (6.3) | 118 (42.8) | x^2^=89.411 Cramer-V=.416 p=.000 |
| No criteria met | 142 (59.2) | 86 (31.2) | x^2^=40.830 Cramer-V=.281 p=.000 |
| **ICD-10 Diagnosis , n (%)** |  |  |  |
| F2 Schizophrenia, schizotypal and delusional disorders | 28 (11.7) | 15 (5.4) | x^2^=6.526 Cramer-V=.112 p=0.11 |
| F21 Schizotypal personality disorder | 10 (4.2) | 0 (0) | x^2^=11.727 Cramer-V=.151 p=.001 |
| F3 Mood (affective) disorders | 51 (21.3) | 90 (32.6) | x^2^=8.340 Cramer-V=.127 p=.004 |
| F4 Neurotic, stress-related and somatoform disorders | 25 (10.4) | 8 (2.9) | x^2^=12.121 Cramer-V=.153 p=.000 |
| F5 Behavioral syndromes associated with physiological disturbances and physical factors | 2 (.8) | 2 (.7) | x^2^=.020 Cramer-V=.006 p=.888 |
| F6 Disorders of adult personality and behavior | 8 (<0.1) | 4 (1.4) | x^2^=2.006 Cramer-V=.062 p=.157 |
| Other current diagnosis, n (%) | 4 (<0.1) | 8 (2.9) | x^2^=.858 Cramer-V=.041 p=.354 |
| ISCED 0-6 = International Standard Classification of Education Level pursuant to the 36^th^ General Conference of the United Nations Educational, Scientific and Cultural Organization ICD-10 = International Classification of Diseases, 10^th^ Revision  ^1^Based on Chi-Quadrat test and Mann-Whitney-U test for distributional discrepancies. **TADS:** Trauma and Distress Scale, **WSS:** Wisconsin Schizotypy Scales. | | | |

| **Table S2:** *Comparison of key features of Schizotypy and Schizotypal personality disorder.[3]** | | |
| --- | --- | --- |
|  | **Schizotypy** | **Schizotypal personality disorder** |
| **Positive traits** | **Magical ideation:** Perceived and/or expressed ideas and beliefs external of the scientific and social consent, though possibly accepted by certain esoteric and spiritual groups | Paranoid beliefs and ideas of reference  Particular beliefs and convictions deviant from norms such as clairvoyance, telepathy and “sixth sense” |
|  | **Perceptive aberration:** Aberrations in the perception oneself and external impulses | Illusions and anomalous perceptions |
|  | Sensory hypersensitivity |  |
| **Negative traits** | **Social anhedonia:** Diminished affect in social situations | Limited number of confidants and legit social contacts  Diminished affect |
|  | Diminished verbal expression | Social anxiety and lack of ability to establish trust with close ones |
|  | Diminished conversational participation due to distorted thinking | Odd thinking and vague speech, diminished ability to express precise diction |
|  | **Physical anhedonia:** Diminished affect in sensory contact and experience |  |
|  | Diminished psychomotor activity |  |
|  | Diminished physical responsiveness and reaction |  |
| * Based on Table 1, Schultze-Lutter F et al. (2019) Psychosis and Schizophrenia-Spectrum Personality Disorders Require Early Detection on Different Symptom Dimensions [3]. | | |

| **Table S3:** *Trauma domains and questions of the Trauma and Distress Scale.* | | |
| --- | --- | --- |
|  | | |
| **Domain** | **Item number** | **Text*** |
| Emotional neglect | Item #5: Item #8: Item #13: Item #21: Item #40: | When I was young, I felt valued or important.  My family were emotionally warm and loving.  When I was young, my family looked after each other.  I respect myself.  My family was supportive and encouraging when I was young. |
| Emotional abuse | Item #10: Item #12: Item #14: Item #26: Item #32: | I felt rejected by my parents / caregivers.  When I was young, I was humiliated by people in my family.  I believe that I am a bad person.  When I was young, I felt hated by a member or members of my family.  I feel that I was put down, criticized and made to feel inferior when I was young. |
| Physical neglect | Item #1: Item #2: Item #4: Item #6: Item #31: | When I was young, I felt safe and protected by somebody.  When I was young, I was often hungry.  I often had to wear ragged or dirty clothes to school.  My parents / caregivers were often drunk, stoned or wasted.  If I needed treatment someone would always take me to see a doctor or nurse when I was young. |
| Physical abuse | Item #9: Item #16: Item #17:  Item #20: Item #24: | When I was young, I was hit so hard that it left marks, cuts or bruises.  I have experienced serious physical assault.  Adults (like teachers, doctors or nurses) noticed cuts, bruises or marks from when I was beaten.  I think I was physically abused when I was young.  I have been involved in life-threatening situations |
| Sexual abuse | Item #22:  Item #25:  Item #30: Item #33: Item #41: | When I was young, someone touched me or tried to make me touch them in a sexual way.  I was forced to keep secrets about someone sexually interfering with me when I was young.  I have experienced sexual assault.  Someone sexually molested me when I was young.  I believe that I was sexually used when I was young. |
| *Possible answers: Never, rarely, sometimes, often, nearly always. | | |

| **Table S4:** *Female sample (n=99) path analysis regression, covariance, variance and r-square data.* | | | | | | | | | | |
| --- | --- | --- | --- | --- | --- | --- | --- | --- | --- | --- |
| **Regressions** | | **Estimate** | | **Std.Err** | | **z-value** | | **P(>\|z\|)** | | **Std.all** |
| **PerAb** | |  | |  | |  | |  | |  |
| EN | | 0.063 | | 0.124 | | 0.509 | | 0.611 | | 0.075 |
| EA | | 0.144 | | 0.112 | | 1.285 | | 0.199 | | 0.184 |
| PN | | 0.124 | | 0.174 | | 0.711 | | 0.477 | | 0.087 |
| PA | | 0.263 | | 0.171 | | 1.534 | | 0.125 | | 0.188 |
| SA | | -0.054 | | 0.084 | | -0.638 | | 0.524 | | -0.068 |
| **MagId** | |  | |  | |  | |  | |  |
| EN | | -0.001 | | 0.113 | | -0.010 | | 0.992 | | -0.002 |
| EA | | 0.034 | | 0.102 | | 0.329 | | 0.742 | | 0.047 |
| PN | | 0.188 | | 0.159 | | 1.181 | | 0.237 | | 0.146 |
| **PA** | | **0.423** | | **0.157** | | **2.699** | | **0.007** | | **0.332** |
| SA | | -0.118 | | 0.077 | | -1.530 | | 0.126 | | -0.164 |
| **PhyAnh** | |  | |  | |  | |  | |  |
| **EN** | | **0.638** | | **0.298** | | **2.138** | | **0.033** | | **0.336** |
| EA | | -0.421 | | 0.270 | | -1.558 | | 0.119 | | -0.239 |
| PN | | -0.491 | | 0.420 | | -1.169 | | 0.243 | | -0.154 |
| PA | | 0.375 | | 0.414 | | 0.907 | | 0.365 | | 0.119 |
| SA | | 0.075 | | 0.203 | | 0.372 | | 0.710 | | 0.042 |
| **SocAnh** | |  | |  | |  | |  | |  |
| **EN** | | **0.487** | | **0.228** | | **2.138** | | **0.033** | | **0.320** |
| EA | | 0.062 | | 0.206 | | 0.299 | | 0.765 | | 0.044 |
| PN | | -0.103 | | 0.320 | | -0.320 | | 0.749 | | -0.040 |
| PA | | 0.238 | | 0.316 | | 0.755 | | 0.450 | | 0.094 |
| SA | | 0.056 | | 0.155 | | 0.359 | | 0.719 | | 0.039 |
| **Covariances:** | | **Estimate** | | **Std.Err** | | **z-value** | | **P(>\|z\|)** | | **Std.all** |
| **PerAb** | |  | |  | |  | |  | |  |
| MagId | | 5.400 | | 1.169 | | 4.618 | | 0.000 | | 0.524 |
| PhyAnh | | 6.347 | | 2.804 | | 2.263 | | 0.024 | | 0.234 |
| SocAnh | | 9.137 | | 2.276 | | 4.014 | | 0.000 | | 0.441 |
| **MagId** | |  | |  | |  | |  | |  |
| PhyAnh | | 2.780 | | 2.514 | | 1.106 | | 0.269 | | 0.112 |
| SocAnh | | 4.080 | | 1.949 | | 2.093 | | 0.036 | | 0.215 |
| **PhyAnh** | |  | |  | |  | |  | |  |
| SocAnh | | 38.672 | | 6.352 | | 6.088 | | 0.000 | | 0.774 |
| **Variances:** | | **Estimate** | | **Std.Err** | | **z-value** | | **P(>\|z\|)** | | **Std.all** |
| PercAb | | 11.264 | | 1.601 | | 7.036 | | 0.000 | | 0.828 |
| MagId | | 9.428 | | 1.340 | | 7.036 | | 0.000 | | 0.837 |
| PhyAnh | | 65.539 | | 9.315 | | 7.036 | | 0.000 | | 0.944 |
| SocAnh | | 38.132 | | 5.420 | | 7.036 | | 0.000 | | 0.856 |
| **R-Square:** | | **Estimate** | |  | |  | |  | |  |
| PercAb | | 0.172 | |  | |  | |  | |  |
| MagId | | 0.163 | |  | |  | |  | |  |
| PhyAnh | | 0.056 | |  | |  | |  | |  |
| SocAnh | | 0.144 | |  | |  | |  | |  |
| EN: emotional neglect, EA: emotional abuse, PN: physical neglect, PA: physical abuse, SA: sexual abuse, PerAb: perceptive aberration, MagId: magic ideation, PhyAnh: physical anhedonia, SocAnh: social anhedonia. Standard Error (Std.Err) all variables standardized (Std.all). Significant regressions highlighted. | | | | | | | | | | |
| **Table S5:** *Male sample (n=141) path analysis regression, covariance, variance and r-square data.* | | | | | | | | | | |
| **Regressions** | **Estimate** | | **Std.Err** | | **z-value** | | **P(>\|z\|)** | | **Std.all** | |
| **PerAb** |  | |  | |  | |  | |  | |
| EN | 0.029 | | 0.096 | | 0.300 | | 0.764 | | 0.037 | |
| EA | 0.028 | | 0.087 | | 0.325 | | 0.745 | | 0.040 | |
| PN | 0.197 | | 0.125 | | 1.576 | | 0.115 | | 0.165 | |
| PA | 0.010 | | 0.135 | | 0.074 | | 0.941 | | 0.008 | |
| **SA** | **0.217** | | **0.107** | | **2.029** | | **0.042** | | **0.190** | |
| **MagId** |  | |  | |  | |  | |  | |
| EN | -0.110 | | 0.105 | | -1.041 | | 0.298 | | -0.130 | |
| **EA** | **0.213** | | **0.096** | | **2.218** | | **0.027** | | **0.274** | |
| PN | 0.124 | | 0.138 | | 0.900 | | 0.368 | | 0.095 | |
| PA | -0.052 | | 0.149 | | -0.347 | | 0.728 | | -0.038 | |
| SA | 0.169 | | 0.118 | | 1.427 | | 0.154 | | 0.134 | |
| **PhyAnh** |  | |  | |  | |  | |  | |
| EN | 0.428 | | 0.267 | | 1.600 | | 0.110 | | 0.206 | |
| EA | -0.067 | | 0.244 | | -0.274 | | 0.784 | | -0.035 | |
| PN | -0.091 | | 0.349 | | -0.261 | | 0.794 | | -0.028 | |
| PA | 0.005 | | 0.378 | | 0.013 | | 0.990 | | 0.001 | |
| SA | 0.079 | | 0.300 | | 0.263 | | 0.793 | | 0.025 | |
| **SocAnh** |  | |  | |  | |  | |  | |
| EN | 0.135 | | 0.208 | | 0.649 | | 0.516 | | 0.082 | |
| EA | 0.075 | | 0.190 | | 0.394 | | 0.694 | | 0.049 | |
| PN | 0.465 | | 0.272 | | 1.712 | | 0.087 | | 0.182 | |
| PA | -0.224 | | 0.294 | | -0.761 | | 0.447 | | -0.083 | |
| SA | 0.258 | | 0.233 | | 1.105 | | 0.269 | | 0.105 | |
| **Covariances:** | **Estimate** | | **Std.Err** | | **z-value** | | **P(>\|z\|)** | | **Std.all** | |
| **PerAb** |  | |  | |  | |  | |  | |
| MagId | 5.150 | | 0.936 | | 5.501 | | 0.000 | | 0.523 | |
| PhyAnh | 0.490 | | 2.105 | | 0.233 | | 0.816 | | 0.020 | |
| SocAnh | 1.833 | | 1.644 | | 1.115 | | 0.265 | | 0.094 | |
| **MagId** |  | |  | |  | |  | |  | |
| PhyAnh | 0.455 | | 2.321 | | 0.196 | | 0.844 | | 0.017 | |
| SocAnh | 2.474 | | 1.817 | | 1.361 | | 0.173 | | 0.115 | |
| **PhyAnh** |  | |  | |  | |  | |  | |
| SocAnh | 39.259 | | 5.647 | | 6.952 | | 0.000 | | 0.722 | |
| **Variances:** | **Estimate** | | **Std.Err** | | **z-value** | | **P(>\|z\|)** | | **Std.all** | |
| PercAb | 8.934 | | 1.064 | | 8.396 | | 0.000 | | 0.903 | |
| MagId | 10.862 | | 1.294 | | 8.396 | | 0.000 | | 0.914 | |
| PhyAnh | 69.880 | | 8.323 | | 8.396 | | 0.000 | | 0.971 | |
| SocAnh | 42.298 | | 5.038 | | 8.396 | | 0.000 | | 0.928 | |
| **R-Square:** | **Estimate** | |  | |  | |  | |  | |
| PercAb | 0.097 | |  | |  | |  | |  | |
| MagId | 0.086 | |  | |  | |  | |  | |
| PhyAnh | 0.029 | |  | |  | |  | |  | |
| SocAnh | 0.072 | |  | |  | |  | |  | |
| EN: emotional neglect, EA: emotional abuse, PN: physical neglect, PA: physical abuse, SA: sexual abuse, PerAb: perceptive aberration, MagId: magic ideation, PhyAnh: physical anhedonia, SocAnh: social anhedonia. Standard Error (Std.Err) all variables standardized (Std.all). Significant regressions highlighted. | | | | | | | | | | |

| **Table S6:** *Total sample (n=240) TADS domains and schizotypy scales intercorrelation.* | | | | | | | |
| --- | --- | --- | --- | --- | --- | --- | --- |
| **TADS** | | | **EN** | **EA** | **PN** | **PA** | **SA** |
|  | **EN** | **r** | 1.000 | **.685** | **.595** | **.329** | .113 |
|  |  | **p** |  | **.000** | **.000** | **.000** | .081 |
|  | **EA** | **r** | **.685** | 1.000 | **.466** | **.492** | **.224** |
|  |  | **p** | **.000** |  | **.000** | **.000** | **.000** |
|  | **PN** | **r** | **.595** | **.466** | 1.000 | **.344** | .046 |
|  |  | **p** | **.000** | **.000** |  | **.000** | .476 |
|  | **PA** | **r** | **.329** | **.492** | **.344** | 1.000 | **.358** |
|  |  | **p** | **.000** | **.000** | **.000** |  | **.000** |
|  | **SA** | **r** | .113 | **.224** | .046 | **.358** | 1.000 |
|  |  | **p** | .081 | **.000** | .476 | **.000** |  |
|  | | | | | | | |
| **WSS** | | | **MagId** | **PerAb** | **PhyAnh** | **SocAnh** | |
|  | **MagId** | **r** | 1.000 | **.535** | .091 | **.225** | |
|  |  | **p** |  | **.000** | .162 | **.000** | |
|  | **PerAb** | **r** | **.535** | 1.000 | .107 | **.275** | |
|  |  | **p** | **.000** |  | .098 | **.000** | |
|  | **PhyAnh** | **r** | .091 | .107 | 1.000 | **.704** | |
|  |  | **p** | .162 | .098 |  | **.000** | |
|  | **SocAnh** | **r** | **.225** | **.275** | **.704** | 1.000 | |
|  |  | **p** | **.000** | **.000** | **.000** |  | |
|  | *p< .05, **p< .00; TADS: Trauma And Distress Scale; WSS: Wisconsin Schizotypy Scales; r: Spearman’s Rho; p: Significance; EN: emotional neglect, EA: emotional abuse, PN: physical neglect, PA: physical abuse, SA: sexual abuse, PerAb: perceptive aberration, MagId: magic ideation, PhyAnh: physical anhedonia, SocAnh: social anhedonia. Significant correlations highlighted. | | | | | | |
|  |  |  |  |  |  |  |  |

| **Table S7:** *Female (n=99) TADS domains and schizotypy scales intercorrelation*. | | | | | | | |
| --- | --- | --- | --- | --- | --- | --- | --- |
| **TADS** | | | **EN** | **EA** | **PN** | **PA** | **SA** |
|  | **EN** | **r** | 1.000 | **.764** | **.602** | **.430** | .131 |
|  |  | **p** |  | **.000** | **.000** | **.000** | .195 |
|  | **EA** | **r** | **.764** | 1.000 | **.500** | **.546** | .196 |
|  |  | **p** | **.000** |  | **.000** | **.000** | .052 |
|  | **PN** | **r** | **.602** | **.500** | 1.000 | **.369** | .072 |
|  |  | **p** | **.000** | **.000** |  | **.000** | .479 |
|  | **PA** | **r** | **.430** | **.546** | **.369** | 1.000 | **.422** |
|  |  | **p** | **.000** | **.000** | **.000** |  | **.000** |
|  | **SA** | **r** | .131 | .196 | .072 | **.422** | 1.000 |
|  |  | **p** | .195 | .052 | .479 | **.000** |  |
|  | | | | | | | |
| **WSS** | | | **MagId** | **PerAb** | **PhyAnh** | **SocAnh** | |
|  | **MagId** | **r** | 1.000 | **.526** | .154 | **.274** | |
|  |  | **p** |  | **.000** | .128 | **.006** | |
|  | **PerAb** | **r** | **.526** | 1.000 | .192 | **.396** | |
|  |  | **p** | **.000** |  | .057 | **.000** | |
|  | **PhyAnh** | **r** | .154 | .192 | 1.000 | **.748** | |
|  |  | **p** | .128 | .057 |  | **.000** | |
|  | **SocAnh** | **r** | **.274** | **.396** | **.748** | 1.000 | |
|  |  | **p** | **.006** | **.000** | **.000** |  | |
|  | *p< .05, **p< .00; TADS: Trauma And Distress Scale; WSS: Wisconsin Schizotypy Scales; r: Spearman’s Rho; p: Significance; EN: emotional neglect, EA: emotional abuse, PN: physical neglect, PA: physical abuse, SA: sexual abuse, PerAb: perceptive aberration, MagId: magic ideation, PhyAnh: physical anhedonia, SocAnh: social anhedonia. Significant correlations highlighted. *p< .05, **p< .00 | | | | | | |

| **Table S8*:*** *Male sample (n=141) TADS domains and schizotypy scales intercorrelation.* | | | | | | | |
| --- | --- | --- | --- | --- | --- | --- | --- |
| **TADS** | | | **EN** | **EA** | **PN** | **PA** | **SA** |
|  | **EN** | **r** | 1.000 | **.627** | **.583** | **.267** | .088 |
|  |  | **p** |  | **.000** | **.000** | **.001** | .302 |
|  | **EA** | **r** | **.627** | 1.000 | **.445** | **.454** | **.215** |
|  |  | **p** | **.000** |  | **.000** | **.000** | **.011** |
|  | **PN** | **r** | **.583** | **.445** | 1.000 | **.328** | .042 |
|  |  | **p** | **.000** | **.000** |  | **.000** | .618 |
|  | **PA** | **r** | **.267** | **.454** | **.328** | 1.000 | **.327** |
|  |  | **p** | **.001** | **.000** | **.000** |  | **.000** |
|  | **SA** | **r** | .088 | **.215** | .042 | **.327** | 1.000 |
|  |  | **p** | .302 | **.011** | .618 | **.000** |  |
|  | | | | | | | |
| **WSS** | | | **MagId** | **PerAb** | **PhyAnh** | **SocAnh** | |
|  | **MagId** | **r** | 1.000 | **.525** | .056 | **.191** | |
|  |  | **p** |  | **.000** | .507 | **.023** | |
|  | **PerAb** | **r** | **.525** | 1.000 | .068 | **.195** | |
|  |  | **p** | **.000** |  | .424 | **.020** | |
|  | **PhyAnh** | **r** | .056 | .068 | 1.000 | **.675** | |
|  |  | **p** | .507 | .424 |  | **.000** | |
|  | **SocAnh** | **r** | **.191** | **.195** | **.675** | 1.000 | |
|  |  | **p** | **.023** | **.020** | **.000** |  | |
|  | *p< .05, **p< .00; TADS: Trauma And Distress Scale; WSS: Wisconsin Schizotypy Scales; r: Spearman’s Rho; p: Significance; EN: emotional neglect, EA: emotional abuse, PN: physical neglect, PA: physical abuse, SA: sexual abuse, PerAb: perceptive aberration, MagId: magic ideation, PhyAnh: physical anhedonia, SocAnh: social anhedonia. Significant correlations highlighted. *p< .05, **p< .00 | | | | | | |
|  |  |  |  |  |  |  |  |

| **Table S9:** *Path analysis code for Open Source “R” version 4.0.2*  library("haven")  library("lavaan")  library("semPlot")  library("dplyr")  n240 <- read_sav("n240.sav")  str(n240)  n99female <- read_sav("n99female.sav")  str(n99female)  n141male <- read_sav("n141male.sav")  str(n141male)  sem1.n240 <-  '  PercAbe_SUM_WSS ~ EmoNeg_SUM_TADS + EmoAb_SUM_TADS + PhyNeg_SUM_TADS + PhyAb_SUM_TADS + SexAb_SUM_TADS  MagIde_SUM_WSS ~ EmoNeg_SUM_TADS + EmoAb_SUM_TADS + PhyNeg_SUM_TADS + PhyAb_SUM_TADS + SexAb_SUM_TADS  PhyAnh_SUM_WSS ~ EmoNeg_SUM_TADS + EmoAb_SUM_TADS + PhyNeg_SUM_TADS + PhyAb_SUM_TADS + SexAb_SUM_TADS  SocAnh_SUM_WSS ~ EmoNeg_SUM_TADS + EmoAb_SUM_TADS + PhyNeg_SUM_TADS + PhyAb_SUM_TADS + SexAb_SUM_TADS  '  Sem1.fit <- sem(sem1.n240, data=n240, estimator="ML")  fitMeasures(sem4.fit, "pnfi")  summary(sem4.fit, fit.measures = TRUE, standardized = TRUE, rsquare = TRUE)  sem2.n99female <-  '  PercAbe_SUM_WSS ~ EmoNeg_SUM_TADS + EmoAb_SUM_TADS + PhyNeg_SUM_TADS + PhyAb_SUM_TADS + SexAb_SUM_TADS  MagIde_SUM_WSS ~ EmoNeg_SUM_TADS + EmoAb_SUM_TADS + PhyNeg_SUM_TADS + PhyAb_SUM_TADS + SexAb_SUM_TADS  PhyAnh_SUM_WSS ~ EmoNeg_SUM_TADS + EmoAb_SUM_TADS + PhyNeg_SUM_TADS + PhyAb_SUM_TADS + SexAb_SUM_TADS  SocAnh_SUM_WSS ~ EmoNeg_SUM_TADS + EmoAb_SUM_TADS + PhyNeg_SUM_TADS + PhyAb_SUM_TADS + SexAb_SUM_TADS  '  sem2.fit <- sem(sem2.n99female, data=n99, estimator="ML")  fitMeasures(sem2.fit, "pnfi")  summary(sem2.fit, fit.measures = TRUE, standardized = TRUE, rsquare = TRUE)  sem3.n141male <-  '  PercAbe_SUM_WSS ~ EmoNeg_SUM_TADS + EmoAb_SUM_TADS + PhyNeg_SUM_TADS + PhyAb_SUM_TADS + SexAb_SUM_TADS  MagIde_SUM_WSS ~ EmoNeg_SUM_TADS + EmoAb_SUM_TADS + PhyNeg_SUM_TADS + PhyAb_SUM_TADS + SexAb_SUM_TADS  PhyAnh_SUM_WSS ~ EmoNeg_SUM_TADS + EmoAb_SUM_TADS + PhyNeg_SUM_TADS + PhyAb_SUM_TADS + SexAb_SUM_TADS  SocAnh_SUM_WSS ~ EmoNeg_SUM_TADS + EmoAb_SUM_TADS + PhyNeg_SUM_TADS + PhyAb_SUM_TADS + SexAb_SUM_TADS  '  sem3.fit <- sem(sem3.n141male, data=n141, estimator="ML")  fitMeasures(sem3.fit, "pnfi")  summary(sem3.fit, fit.measures = TRUE, standardized = TRUE, rsquare = TRUE) |
| --- |
